# Tetra-μ_2_-acetato-tetraaquadi-μ_3_-oxido-octaoxidotetrauranium(VI) methanol disolvate tetrahydrate

**DOI:** 10.1107/S1600536811050549

**Published:** 2011-11-30

**Authors:** Hisham Sleem, Paris Georghiou, Louise N. Dawe

**Affiliations:** a Department of Chemistry, Memorial University of Newfoundland, St. Johns, NL, A1B 3X7, Canada; b Department of Chemistry and Centre for Chemical Analysis, Research and Training (C-CART), X-Ray Diffraction Laboratory, Memorial University of Newfoundland, St. Johns, NL, A1B 3X7, Canada

## Abstract

The centrosymmetric title tetra­mer, [U_4_(C_2_H_3_O_2_)_4_O_10_(H_2_O)_4_]·2CH_4_O, has a near planar core [maximum deviation from the least squares plane of 0.294 (6) Å]. It consists of two hexa­gonal–bipyramidally coordinated U^VI^ atoms connected *via* μ_2_-O (acetate) and μ_3_-O (oxide) bridges in the equatorial plane to two penta­gonal–bipyramidally coordinated U^VI^ atoms. The equatorial plane of each U^VI^ atom is completed by a bound water mol­ecule, while the axial positions are occupied by uranyl (UO_2_)^2+^ O atoms. Multiple O—H⋯O hydrogen bonds are present, including a lattice methanol mol­ecule bound to one of the penta­gonal bipyramidal uranyl O atoms, as well as two different *C* ^1^_1_(6) chains orginating from a donor water mol­ecule, *via* a uranyl oxygen acceptor and an acetate acceptor on different, adjacent tetra­mers. Finally, the unit cell contains four U^VI^ tetra­mers, all connected by hydrogen bonding, forming a supra­molecular *R* ^4^_4_(24) ring.

## Related literature

For structurally similar tetra­meric complexes with U^VI^, *M*_4_[(UO_2_)_4_(μ_3_-O)_2_ *L*_4_] (*M* = NH_4_^+^, K^+^, Cs^+^; *L* = phthalate), see: Charushnikova *et al.* (2005 ▶), and with Bi, [Bi_2_(μ_3_-O)(OCH(CF_3_)_2_)_2_(μ-OCH(CF_3_)_2_)_2_(Solv)]_2_ (Solv = C_7_H_8_, Et_2_O, thf), see: Andrews *et al.* (2008 ▶). For a planar, mixed valent U^V^_2_U^VI^_2_ alkoxide, see: Zozulin *et al.* (1982 ▶). For a *p*-benzyl­calix[7]arene complex containing a hexa­nuclear U^VI^ cluster with a planar tetra­meric core, see: Thuéry *et al.* (1999 ▶), and for dinuclear uranyl-containing salen [*N*,*N*′-ethyl­enebis(salicyl­imine)] complexes, see: Amato *et al.* (2007 ▶). For bond-valence-sum calculations, see: Wills (2010 ▶). 
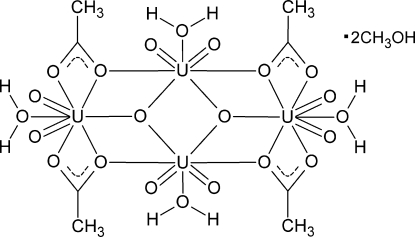


## Experimental

### 

#### Crystal data

[U_4_(C_2_H_3_O_2_)_4_O_10_(H_2_O)_4_]·2CH_4_O
*M* *_r_* = 1484.44
Monoclinic, 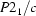

*a* = 8.334 (3) Å
*b* = 10.649 (3) Å
*c* = 16.763 (5) Å
β = 107.632 (4)°
*V* = 1417.8 (8) Å^3^
*Z* = 2
Mo *K*α radiation
μ = 22.87 mm^−1^
*T* = 163 K
0.10 × 0.07 × 0.05 mm

#### Data collection

Rigaku Saturn70 CCD diffractometer
Absorption correction: numerical (*ABSCOR*; Higashi, 1999 ▶) *T*_min_ = 0.638, *T*_max_ = 0.880
15149 measured reflections
3255 independent reflections
3136 reflections with *I* > 2σ(*I*)
*R*_int_ = 0.076

#### Refinement

*R*[*F* ^2^ > 2σ(*F* ^2^)] = 0.034
*wR*(*F* ^2^) = 0.088
*S* = 1.09
3255 reflections
188 parameters
6 restraints
H atoms treated by a mixture of independent and constrained refinement
Δρ_max_ = 1.71 e Å^−3^
Δρ_min_ = −2.85 e Å^−3^

### 

Data collection: *CrystalClear-SM Expert* (Rigaku, 2009 ▶); cell refinement: *CrystalClear-SM Expert*; data reduction: *CrystalClear-SM Expert*; program(s) used to solve structure: *SHELXS97* (Sheldrick, 2008 ▶); program(s) used to refine structure: *SHELXL97* (Sheldrick, 2008 ▶); molecular graphics: *Mercury* (Macrae *et al.*, 2006 ▶); software used to prepare material for publication: *publCIF* (Westrip, 2010 ▶).

## Supplementary Material

Crystal structure: contains datablock(s) I, global. DOI: 10.1107/S1600536811050549/br2182sup1.cif

Structure factors: contains datablock(s) I. DOI: 10.1107/S1600536811050549/br2182Isup2.hkl

Additional supplementary materials:  crystallographic information; 3D view; checkCIF report

## Figures and Tables

**Table 1:** Hydrogen-bond geometry (Å, °)

*D*—H⋯*A*	*D*—H	H⋯*A*	*D*⋯*A*	*D*—H⋯*A*
O12—H12⋯O9	0.84	2.31	3.009 (10)	141
O8—H8*A*⋯O7^i^	0.87 (6)	2.07 (6)	2.859 (8)	151 (7)
O8—H8*B*⋯O12^ii^	0.88 (7)	1.78 (6)	2.645 (8)	170 (9)
O11—H11*A*⋯O10^iii^	0.87 (7)	1.88 (7)	2.736 (7)	166 (8)
O11—H11*B*⋯O2^iv^	0.88 (7)	1.83 (7)	2.705 (7)	173 (7)

Symmetry codes: (i) 
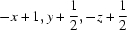
; (ii) 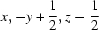; (iii) 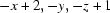; (iv) 
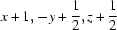
.

**Fig. 1.**
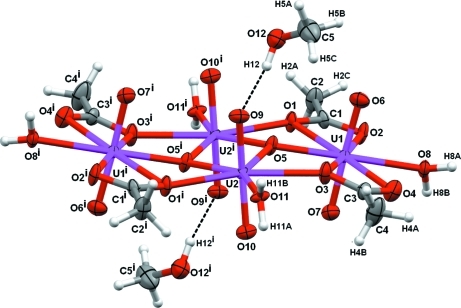
50% displacement ellipsoid representation of the title compound; hydrogen bonding indicated by dashed lines. Symmetry code: (i) -x + 1, -y, -z + 1.

**Fig. 2.**
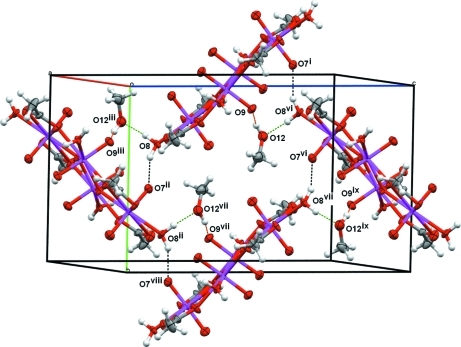
50% displacement ellipsoid representation of the packed unit cell for the title compound; hydrogen bonding indicated by dashed lines. H-bonding for O11—H11A···O10iv and O11—H11B···O2v not shown. Symmetry codes: (ii)-x + 1, y + 1/2, -z + 1/2, (vi) x, -y + 1/2, z + 1/2, (vii) -x + 1, -y + 1, -z + 1, (viii)x, y + 1, z, (ix) -x + 1, y + 1/2, -z + 3/2.

## supplementary crystallographic information

### Comment

In connection with our on-going studies on metal-binding properties of a new series of macrocylic polyamide compounds, we were interested in determining whether such macrocycles, by analogy with analogous salen (N,N'-Ethylenebis(salicylimine)) compounds, would also form stable uranyl complexes. Using similar conditions to those which Amato *et al.* (2007) employed with their salens, the title compound crystallized out in the form reported herein.

Bond valence sum calculations (Wills, 2010) performed on the title compound indicate that both crystallographically independent uranium atoms are in the +6 oxidations state (U1 = 6.095, U2 = 6.074). The centrosymmetric U(VI) tetramer consists of two hexagonal bipyramids, and two pentagonal bipyramids (U1 and U2, and symmetry equivalents (-*x* + 1, -*y*, -*z* + 1), respectively; Figure 1), with (UO_2_)^2+^ oxygen-atoms occupying the axial positions for both U1 and U2. For U1, the equatorial plane consists of *trans*-bidentate acetate anions, each with one bridging µ_2_-O-atom (O1 and O3), and a µ_3_-O^2-^ anion (O5) *trans* to a water molecule. The equatorial plane of U2 is therefore composed of the aforementioned µ_2_-O and µ_3_-O atoms, and their inversion-symmetry generated counter-parts, as well as water molecule (O11). Similar to the description given by Andrews *et al.* (2008) for [Bi_2_(µ_3_-O)(OCH(CF_3_)_2_)_2_(µ-OCH(CF_3_)_2_)_2_(Solv)]_2_ (Solv = C_7_H_8_, Et_2_O, thf) tetramers, this complex consists of a near planar, ten atom "raft", with maximum deviation from the least squares plane [U1, U2, O1–5 and symmetry equivalents (-*x* + 1, -*y*, -*z* + 1)] of 0.294 (6) Å for O5. Examination of longer range interactions reveals numerous hydrogen bonds, including a lattice solvent methanol molecule bound to one of the pentagonal bipyramidal uranyl oxygen atoms (O12—H12···O9; Figure 1), which further bridges to a bound water molecule of a second tetramer (O8—H8B···O12^iii^, (iii) *x*, -*y*+1/2, *z*-1/2; Figure 2, green dashed lines.) The tetramers interact *via* an additional hydrogen bond, wherby the aforementioned water molecule acts as a donor to one of the hexagonal bipyramidal uranyl oxygen atoms on the first assembly (O8—H8A···O7^ii^, (ii) -*x*+1, *y*+1/2, -*z*+1/2; Figure 2, black dashed lines) leading to a 24-membered H-bonded ring (graph set notation *R*^4^_4_(24)) which spans all four tetramers present in the unit cell.

### Experimental

160 mg (0.337 mmol) of uranyl acetate dihydrate (UO_2_(CH_3_COO)_2_.2H_2_O) was dissolved in 1 ml methanol, and warmed in a hot water bath (323 K) for 5 minutes. The solution was left at room temperature (293 K) for slow evaporation. Yellow prismatic crystals, suitable for analysis by X-ray diffraction, formed after one week.

### Refinement

The water H-atoms, H8(A,B) and H11(A,B), were located from difference Fourier maps, and were refined with distance and angle restraints: O—H = 0.87 (2) Å, H—O—H = 104.50 (4)°. The C-bound methyl and methanolic H-atoms were included in calculated positions and treated as riding atoms: *X*—H = 0.98 and 0.84 Å for *H*-methyl and H-OMe H-atoms, respectively. For all H atoms, *U*_iso_(H) = k × *U*_eq_(parent atom), where k = 1.2 for *H*-methyl and k = 1.5 for all O-bound H-atoms. The final electron density synthesis shows the highest peak of 1.71 eÅ^3^ located 0.98 Å from U2 and the deepest hole of -2.85 eÅ^3^ located 0.72 Å from U1 and may be the result of residual absorption effects.

### Crystal data

**Table:**

[U_4_(C_2_H_3_O_2_)_4_O_10_(H_2_O)_4_]·2CH_4_O	*F*(000) = 1296
*M**_r_* = 1484.44	*D*_x_ = 3.477 Mg m^−^^3^
Monoclinic, *P*2_1_/*c*	Mo *K*α radiation, λ = 0.71075 Å
Hall symbol: -P 2ybc	Cell parameters from 4946 reflections
*a* = 8.334 (3) Å	θ = 1.9–29.5°
*b* = 10.649 (3) Å	µ = 22.87 mm^−^^1^
*c* = 16.763 (5) Å	*T* = 163 K
β = 107.632 (4)°	Prism, yellow
*V* = 1417.8 (8) Å^3^	0.10 × 0.07 × 0.05 mm
*Z* = 2	

### Data collection

**Table:**

Rigaku Saturn70 CCD diffractometer	3255 independent reflections
Radiation source: fine-focus sealed tube	3136 reflections with *I* > 2σ(*I*)
graphite - Rigaku SHINE	*R*_int_ = 0.076
Detector resolution: 14.63 pixels mm^-1^	θ_max_ = 27.5°, θ_min_ = 3.1°
ω scans	*h* = −10→10
Absorption correction: numerical (*ABSCOR*; Higashi, 1999)	*k* = −13→13
*T*_min_ = 0.638, *T*_max_ = 0.880	*l* = −21→21
15149 measured reflections	

### Refinement

**Table:**

Refinement on *F*^2^	Primary atom site location: structure-invariant direct methods
Least-squares matrix: full	Secondary atom site location: difference Fourier map
*R*[*F*^2^ > 2σ(*F*^2^)] = 0.034	Hydrogen site location: inferred from neighbouring sites
*wR*(*F*^2^) = 0.088	H atoms treated by a mixture of independent and constrained refinement
*S* = 1.09	*w* = 1/[σ^2^(*F*_o_^2^) + (0.0469*P*)^2^ + 6.6005*P*] where *P* = (*F*_o_^2^ + 2*F*_c_^2^)/3
3255 reflections	(Δ/σ)_max_ = 0.001
188 parameters	Δρ_max_ = 1.71 e Å^−^^3^
6 restraints	Δρ_min_ = −2.85 e Å^−^^3^

### Special details

**Table:**

Geometry. All e.s.d.'s (except the e.s.d. in the dihedral angle between two l.s. planes) are estimated using the full covariance matrix. The cell e.s.d.'s are taken into account individually in the estimation of e.s.d.'s in distances, angles and torsion angles; correlations between e.s.d.'s in cell parameters are only used when they are defined by crystal symmetry. An approximate (isotropic) treatment of cell e.s.d.'s is used for estimating e.s.d.'s involving l.s. planes.
Refinement. Refinement of *F*^2^ against ALL reflections. The weighted *R*-factor *wR* and goodness of fit *S* are based on *F*^2^, conventional *R*-factors *R* are based on *F*, with *F* set to zero for negative *F*^2^. The threshold expression of *F*^2^ > σ(*F*^2^) is used only for calculating *R*-factors(gt) *etc*. and is not relevant to the choice of reflections for refinement. *R*-factors based on *F*^2^ are statistically about twice as large as those based on *F*, and *R*- factors based on ALL data will be even larger.

### Fractional atomic coordinates and isotropic or equivalent isotropic displacement parameters (Å^2^)

**Table:**

	*x*	*y*	*z*	*U*_iso_*/*U*_eq_	
U1	0.41912 (3)	0.20869 (2)	0.332933 (15)	0.01529 (10)	
U2	0.71490 (3)	0.06455 (2)	0.542406 (14)	0.01236 (10)	
O1	0.1576 (7)	0.0796 (5)	0.3408 (3)	0.0247 (11)	
O2	0.1274 (7)	0.1931 (5)	0.2295 (3)	0.0272 (12)	
O3	0.7084 (7)	0.2370 (6)	0.4457 (4)	0.0296 (13)	
O4	0.6738 (7)	0.3262 (6)	0.3266 (4)	0.0306 (13)	
O5	0.4581 (6)	0.0936 (5)	0.4484 (3)	0.0217 (11)	
O6	0.3486 (7)	0.3399 (5)	0.3788 (4)	0.0277 (12)	
O7	0.4880 (7)	0.0816 (5)	0.2841 (4)	0.0277 (12)	
O8	0.3755 (7)	0.3336 (5)	0.2040 (3)	0.0230 (11)	
H8A	0.403 (12)	0.406 (4)	0.188 (5)	0.034*	
H8B	0.367 (13)	0.286 (6)	0.160 (4)	0.034*	
O9	0.6588 (7)	0.1628 (5)	0.6159 (4)	0.0264 (12)	
O10	0.7947 (6)	−0.0283 (5)	0.4734 (3)	0.0236 (11)	
O11	0.9960 (6)	0.1499 (5)	0.5992 (3)	0.0202 (10)	
H11A	1.075 (7)	0.121 (8)	0.580 (5)	0.030*	
H11B	1.047 (9)	0.200 (7)	0.641 (4)	0.030*	
O12	0.3208 (9)	0.2904 (6)	0.5615 (4)	0.0376 (15)	
H12	0.3876	0.2346	0.5557	0.056*	
C1	0.0629 (9)	0.1183 (7)	0.2696 (4)	0.0207 (14)	
C2	−0.1168 (10)	0.0795 (8)	0.2383 (6)	0.0332 (19)	
H2A	−0.1601	0.0663	0.2859	0.040*	
H2B	−0.1261	0.0013	0.2064	0.040*	
H2C	−0.1825	0.1454	0.2020	0.040*	
C3	0.7675 (10)	0.2993 (7)	0.3966 (5)	0.0239 (16)	
C4	0.9472 (12)	0.3420 (11)	0.4242 (7)	0.049 (3)	
H4A	0.9639	0.4053	0.3850	0.059*	
H4B	1.0212	0.2701	0.4252	0.059*	
H4C	0.9741	0.3785	0.4803	0.059*	
C5	0.4006 (16)	0.4061 (10)	0.5715 (6)	0.051 (3)	
H5A	0.4034	0.4418	0.6258	0.062*	
H5B	0.3388	0.4628	0.5267	0.062*	
H5C	0.5159	0.3955	0.5692	0.062*	

### Atomic displacement parameters (Å^2^)

**Table:**

	*U*^11^	*U*^22^	*U*^33^	*U*^12^	*U*^13^	*U*^23^
U1	0.01397 (15)	0.01724 (15)	0.01456 (16)	0.00003 (8)	0.00417 (11)	0.00355 (8)
U2	0.00972 (14)	0.01612 (15)	0.01148 (15)	−0.00104 (8)	0.00357 (10)	0.00029 (8)
O1	0.018 (3)	0.031 (3)	0.021 (3)	−0.001 (2)	0.000 (2)	0.014 (2)
O2	0.023 (3)	0.037 (3)	0.018 (3)	−0.006 (2)	0.002 (2)	0.015 (2)
O3	0.020 (3)	0.034 (3)	0.033 (3)	−0.006 (2)	0.005 (2)	0.016 (3)
O4	0.022 (3)	0.043 (3)	0.025 (3)	−0.007 (2)	0.003 (2)	0.005 (2)
O5	0.012 (2)	0.031 (3)	0.019 (3)	−0.006 (2)	0.0002 (19)	0.010 (2)
O6	0.019 (3)	0.032 (3)	0.029 (3)	0.006 (2)	0.003 (2)	−0.001 (2)
O7	0.026 (3)	0.029 (3)	0.029 (3)	0.002 (2)	0.011 (2)	−0.001 (2)
O8	0.023 (3)	0.025 (3)	0.022 (3)	0.001 (2)	0.008 (2)	0.011 (2)
O9	0.021 (3)	0.029 (3)	0.032 (3)	0.000 (2)	0.011 (2)	−0.008 (2)
O10	0.016 (2)	0.032 (3)	0.022 (3)	−0.004 (2)	0.004 (2)	−0.009 (2)
O11	0.014 (2)	0.027 (3)	0.022 (3)	−0.006 (2)	0.009 (2)	−0.010 (2)
O12	0.042 (4)	0.040 (4)	0.033 (3)	0.004 (3)	0.014 (3)	0.004 (3)
C1	0.017 (3)	0.020 (3)	0.020 (3)	0.000 (3)	−0.001 (3)	0.006 (3)
C2	0.016 (4)	0.043 (5)	0.036 (5)	−0.006 (3)	0.001 (3)	0.014 (4)
C3	0.023 (4)	0.021 (3)	0.031 (4)	−0.005 (3)	0.012 (3)	0.003 (3)
C4	0.028 (5)	0.073 (7)	0.044 (6)	−0.014 (5)	0.006 (4)	0.021 (5)
C5	0.072 (8)	0.041 (5)	0.032 (5)	0.009 (5)	0.002 (5)	−0.005 (4)

### Geometric parameters (Å, °)

**Table:**

U1—O7	1.765 (6)	O4—C3	1.230 (10)
U1—O6	1.779 (6)	O5—U2^i^	2.252 (5)
U1—O5	2.230 (5)	O8—H8A	0.87 (2)
U1—O8	2.469 (5)	O8—H8B	0.87 (2)
U1—O4	2.494 (6)	O11—H11A	0.87 (2)
U1—O2	2.528 (6)	O11—H11B	0.87 (2)
U1—O3	2.592 (6)	O12—C5	1.385 (12)
U1—O1	2.614 (5)	O12—H12	0.8400
U2—O9	1.784 (5)	C1—C2	1.487 (10)
U2—O10	1.795 (5)	C2—H2A	0.9800
U2—O5^i^	2.252 (5)	C2—H2B	0.9800
U2—O5	2.261 (5)	C2—H2C	0.9800
U2—O11	2.422 (5)	C3—C4	1.498 (12)
U2—O3	2.438 (5)	C4—H4A	0.9800
U2—O1^i^	2.461 (5)	C4—H4B	0.9800
O1—C1	1.284 (8)	C4—H4C	0.9800
O1—U2^i^	2.461 (5)	C5—H5A	0.9800
O2—C1	1.262 (9)	C5—H5B	0.9800
O3—C3	1.268 (9)	C5—H5C	0.9800
			
O7—U1—O6	178.0 (3)	O5—U2—O1^i^	136.78 (17)
O7—U1—O5	89.9 (2)	O11—U2—O1^i^	77.77 (18)
O6—U1—O5	92.1 (2)	O3—U2—O1^i^	156.28 (18)
O7—U1—O8	89.4 (2)	C1—O1—U2^i^	159.4 (5)
O6—U1—O8	88.6 (2)	C1—O1—U1	94.1 (4)
O5—U1—O8	179.2 (2)	U2^i^—O1—U1	101.70 (18)
O7—U1—O4	88.0 (2)	C1—O2—U1	98.8 (4)
O6—U1—O4	91.2 (2)	C3—O3—U2	153.0 (5)
O5—U1—O4	114.24 (18)	C3—O3—U1	92.6 (5)
O8—U1—O4	66.05 (18)	U2—O3—U1	103.06 (19)
O7—U1—O2	90.7 (2)	C3—O4—U1	98.3 (5)
O6—U1—O2	88.5 (2)	U1—O5—U2^i^	122.8 (2)
O5—U1—O2	114.60 (17)	U1—O5—U2	122.6 (2)
O8—U1—O2	65.10 (17)	U2^i^—O5—U2	109.9 (2)
O4—U1—O2	131.14 (18)	U1—O8—H8A	140 (6)
O7—U1—O3	93.8 (2)	U1—O8—H8B	112 (5)
O6—U1—O3	87.1 (2)	H8A—O8—H8B	102 (4)
O5—U1—O3	64.64 (17)	U2—O11—H11A	118 (5)
O8—U1—O3	115.72 (17)	U2—O11—H11B	136 (5)
O4—U1—O3	50.00 (18)	H11A—O11—H11B	105 (4)
O2—U1—O3	175.5 (2)	C5—O12—H12	109.5
O7—U1—O1	90.8 (2)	O2—C1—O1	117.2 (6)
O6—U1—O1	90.1 (2)	O2—C1—C2	122.2 (7)
O5—U1—O1	64.63 (17)	O1—C1—C2	120.5 (7)
O8—U1—O1	115.07 (17)	C1—C2—H2A	109.5
O4—U1—O1	178.31 (18)	C1—C2—H2B	109.5
O2—U1—O1	49.97 (16)	H2A—C2—H2B	109.5
O3—U1—O1	129.03 (16)	C1—C2—H2C	109.5
O9—U2—O10	173.7 (2)	H2A—C2—H2C	109.5
O9—U2—O5^i^	94.8 (2)	H2B—C2—H2C	109.5
O10—U2—O5^i^	90.1 (2)	O4—C3—O3	118.9 (7)
O9—U2—O5	90.6 (2)	O4—C3—C4	120.7 (7)
O10—U2—O5	94.8 (2)	O3—C3—C4	120.4 (8)
O5^i^—U2—O5	70.1 (2)	C3—C4—H4A	109.5
O9—U2—O11	86.2 (2)	C3—C4—H4B	109.5
O10—U2—O11	87.5 (2)	H4A—C4—H4B	109.5
O5^i^—U2—O11	144.73 (18)	C3—C4—H4C	109.5
O5—U2—O11	145.17 (18)	H4A—C4—H4C	109.5
O9—U2—O3	93.4 (2)	H4B—C4—H4C	109.5
O10—U2—O3	85.7 (2)	O12—C5—H5A	109.5
O5^i^—U2—O3	136.25 (19)	O12—C5—H5B	109.5
O5—U2—O3	66.93 (18)	H5A—C5—H5B	109.5
O11—U2—O3	78.65 (18)	O12—C5—H5C	109.5
O9—U2—O1^i^	87.5 (2)	H5A—C5—H5C	109.5
O10—U2—O1^i^	90.8 (2)	H5B—C5—H5C	109.5
O5^i^—U2—O1^i^	67.08 (17)		

Symmetry codes: (i) −*x*+1, −*y*, −*z*+1.

### Hydrogen-bond geometry (Å, °)

**Table:**

*D*—H···*A*	*D*—H	H···*A*	*D*···*A*	*D*—H···*A*
O12—H12···O9	0.84	2.31	3.009 (10)	141
O12—H12···O5	0.84	2.54	3.257 (9)	143
O8—H8A···O7^ii^	0.87 (6)	2.07 (6)	2.859 (8)	151 (7)
O8—H8B···O12^iii^	0.88 (7)	1.78 (6)	2.645 (8)	170 (9)
O11—H11A···O10^iv^	0.87 (7)	1.88 (7)	2.736 (7)	166 (8)
O11—H11B···O2^v^	0.88 (7)	1.83 (7)	2.705 (7)	173 (7)

Symmetry codes: (ii) −*x*+1, *y*+1/2, −*z*+1/2; (iii) *x*, −*y*+1/2, *z*−1/2; (iv) −*x*+2, −*y*, −*z*+1; (v) *x*+1, −*y*+1/2, *z*+1/2.
